# From the INVICTUS Trial to Current Considerations: It’s Not Time to Retire Vitamin K Inhibitors Yet!

**DOI:** 10.3390/ph17111459

**Published:** 2024-10-31

**Authors:** Akshyaya Pradhan, Somya Mahalawat, Marco Alfonso Perrone

**Affiliations:** 1 Department of Cardiology, King George’s Medical University, Lucknow 226003, India; somyamahalawat@ymail.com; 2 Division of Cardiology and CardioLab, Department of Clinical Sciences and Translational Medicine, University of Rome Tor Vergata, 00133 Rome, Italy

**Keywords:** oral anticoagulants, valvular heart disease, mitral stenosis, DOAC, VKA

## Abstract

Atrial fibrillation (AF) is a common arrhythmia in clinical practice, and oral anticoagulation is the cornerstone of stroke prevention in AF. Direct oral anticoagulants (DOAC) significantly reduce the incidence of intracerebral hemorrhage with preserved efficacy for preventing stroke compared to vitamin K antagonists (VKA). However, the pivotal randomized controlled trials (RCTs) of DOAC excluded patients with valvular heart disease, especially mitral stenosis, which remains an exclusion criterion for DOAC use. The INVICTUS study was a large multicenter global RCT aimed at evaluating the role of DOAC compared to VKA in stroke prevention among patients with rheumatic valvular AF. In this study, rivaroxaban failed to prove superiority over VKA in preventing the composite primary efficacy endpoints of stroke, systemic embolism, myocardial infarction, and death. Unfortunately, the bleeding rates were not lower with rivaroxaban either. The death and drug discontinuation rates were higher in the DOAC arm. Close to the heels of the dismal results of INVICTUS, an apixaban trial in prosthetic heart valves, PROACT-Xa, was also prematurely terminated due to futility. Hence, for AF complicating moderate-to-severe mitral stenosis or prosthetic valve VKA remains the standard of care. However, DOAC can be used in patients with surgical bioprosthetic valve implantation, TAVR, and other native valve diseases with AF, except for moderate-to-severe mitral stenosis. Factor XI inhibitors represent a breakthrough in anticoagulation as they aim to dissociate thrombosis from hemostasis, thereby indicating a potential to cut down bleeding further. Multiple agents (monoclonal antibodies—e.g., osocimab, anti-sense oligonucleotides—e.g., fesomersen, and small molecule inhibitors—e.g., milvexian) have garnered positive data from phase II studies, and many have entered the phase III studies in AF/Venous thromboembolism. Future studies on conventional DOAC and new-generation DOAC will shed further light on whether DOAC can dethrone VKA in valvular heart disease.

## 1. Introduction

Atrial fibrillation (AF) is a common arrhythmia in clinical practice and carries a high risk of stroke/systemic embolism [1]. Oral anticoagulation (OAC) has been the cornerstone of stroke prevention in patients with AF and vitamin k antagonists have been the sheet anchor for OAC for the past 5 decades. However, their use is plagued with multiple shortcomings, such as drug interactions, food interactions, frequent INR monitoring, a narrow therapeutic range, and the risk of intracerebral hemorrhage [1]. Direct oral anticoagulants (DOAC) represent a significant advancement in OAC for AF without the need for frequent INR monitoring and with minimal food/drug interaction. More significantly, they reduce the incidence of intracerebral hemorrhage with preserved efficacy for preventing stroke compared to vitamin K antagonists (VKA), as evidenced in four large pivotal RCTs involving AF patients [2]. However, most of these trials excluded patients with valvular heart disease, especially mitral stenosis, which remains an exclusion criterion for DOAC use. INVICTUS was a large multicenter global RCT aimed at evaluating the role of DOAC compared to VKA in stroke prevention among patients with rheumatic valvular AF. In this review, we first discuss the INVICTUS study and then provide the current perspectives in the background of the results of other studies of DOAC in valvular heart disease.

## 2. The INVICTUS Trial

INVICTUS was an international multicenter, double-blind trial in patients with rheumatic mitral valve disease [3]. In this trial, once-daily rivaroxaban (at a dose of 20 or 15 mg, according to renal function) was compared with a dose-adjusted vitamin K antagonist in patients with documented rheumatic mitral valve disease and atrial fibrillation (AF). The primary efficacy outcome was a composite of total stroke and systemic embolism. The secondary outcomes were acute myocardial infarction and death from vascular (cardiac or non-cardiac) causes. The primary safety outcome was major bleeding as defined by the International Society of Thrombosis and Hemostasis (ISTH) [3].

The trial was open-label, double-blinded, and used the intention-to-treat principle for the efficacy analysis. The included patients were aged ≥18 years and had echocardiography-proven rheumatic heart disease with documented atrial fibrillation or atrial flutter at any time. Additionally, they were required to have at least one of the following criteria: a CHA_2_DS_2_VASc score of at least 2, mitral stenosis with a mitral valve area <2 cm^2^, or echocardiographic evidence of either left atrial spontaneous echo contrast or left atrial thrombus.

The trial excluded patients with a mechanical heart valve or with the likelihood of receiving one within the next 6 months, the use of dual antiplatelet therapy, treatment with dual strong inhibitors of CYP3A4 and P-glycoprotein, and the presence of severe renal disease (estimated GFR filtration rate < 15 mL/min). Women of childbearing age were excluded if they were pregnant or not using contraception.

**Figure 1:**
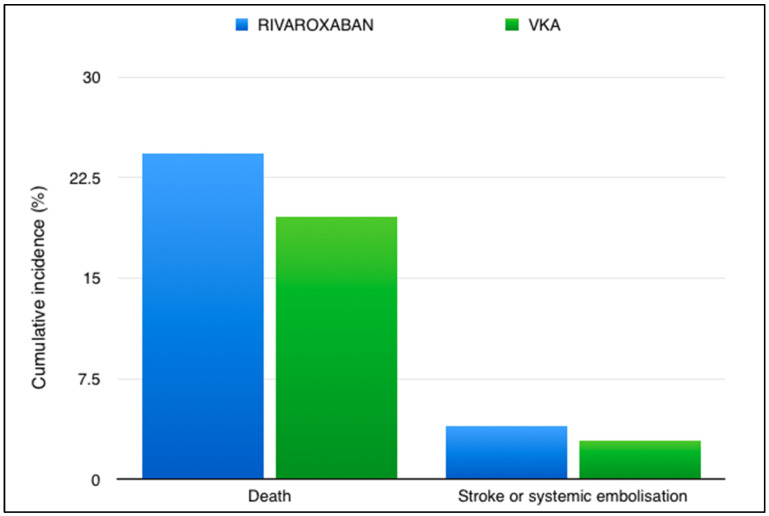
Rates of events in the INVICTUS trial in the Rivaroxaban and VKA arms. [VKA—Vitamin K antagonist].

Between August 2016 and September 2019, 4565 patients were enrolled from 138 trial sites across 24 countries. On 4 February 2022, the data and safety monitoring board recommended that the trial be terminated because the primary question addressed by the trial had been answered satisfactorily. The primary endpoint (composite of stroke, systemic embolism, myocardial infarction, or death from vascular or unknown causes) transpired in 560 of 2275 patients in the rivaroxaban group and in 446 of 2256 patients in the VKA group (proportional-hazards ratio, 1.25; 95% confidence interval [CI], 1.10 to 1.41). The restricted mean survival time was 1599 days in the rivaroxaban group and 1675 days in the VKA group (difference −76 days; 95% CI, −121 to −31 days; *p* < 0.001 for superiority). More patients in the rivaroxaban group than in the VKA group had a stroke (90 vs. 65 patients), a finding that was almost entirely driven by the higher rate of ischemic stroke in the rivaroxaban group (Figure 1). A total of 552 patients in the rivaroxaban group and 442 patients in the VKA group died (difference in restricted mean survival time, −72 days; 95% CI, −117 to −28). The difference in mortality was almost entirely due to the lower rates of sudden cardiac death and death due to mechanical or pump failure in the VKA group than in the rivaroxaban group. Between-group differences in the rates of stroke and death were similar in the on-treatment and intention-to-treat analyses. The rates of major bleeding did not differ significantly between the treatment groups.

## 3. Implications for Practice

AF may occur because of diverse pathophysiological conditions that lead to remodeling of the left atrium. Patients with atrial fibrillation are at an increased risk for embolic stroke owing to the formation of a thrombus in the left atrium, which can embolize, leading to stroke or systemic embolism [4]. Atrial fibrillation affects >30 million people worldwide. It increases the risk of stroke 5-fold and is thought to cause 15% of all strokes. Most patients with AF receive lifelong oral anticoagulation therapy to prevent strokes or systemic embolisms. During the past decade, DOAC has proven to be superior or non-inferior to warfarin for stroke prevention in non-valvular AF (NVAF), both in large randomized controlled trials and real-world observational studies, and is now the first-choice recommendation for oral anticoagulation in contemporary guidelines in preference to VKA [1,5]. Nonetheless, VKA remains the drug of choice in some conditions, such as mechanical valve prostheses, including in pregnant patients, and rheumatic mitral stenosis.

The advantage of DOAC over VKA is primarily due to their high efficacy in averting stroke in NVAF, with a lower incidence of major bleeding, especially intracranial hemorrhage. Additional advantages include ease of use, minor or no drug and food interactions, predictable pharmacokinetics and pharmacodynamics, rapid onset and offset of action, shorter half-life, and obviation of the need for strict laboratory monitoring. However, DOAC has some disadvantages. DOAC remains like higher cost, specific antidote unavailability, and limited experience with these drugs outside the purview of NVAF (Table 1). In addition, DOAC is contraindicated in patients with severe renal and hepatic disease (absence of validated monitoring tests), patients with mechanical heart valves, individuals younger than 18 years of age, and antiphospholipid antibody syndrome; however, there is a high demand for DOAC because of the convenience of administration for caregivers and patients alike [6]. On the contrary, despite regular compliance, more than half of the patients on VKA therapy fail to maintain their international normalized ratio (INR) in the therapeutic range, leading to a risk potential for thromboembolic events.

Rheumatic mitral valve disease continues to be a burden in low-income and middle-income countries [7]. It predominantly affects poor socio-economic strata who have poor access to healthcare facilities for myriad reasons. Because the seminal trials of DOAC excluded moderate to severe mitral stenosis, VKA remains the standard of care for rheumatic mitral valve disease with AF. Recent data also suggest overestimation of AF risk in rhematic mitral stenosis due to flawed interpretation of data [8]. Moreover, such patients are younger by a decade or two with a paucity of conventional cardiovascular risk factors compared to the typical AF population enrolled in seminal DOAC trials. Hence, the INVICTUS trial was poised to answer the clinical question of whether we can safely replace VKA with DOAC in this setting of rheumatic mitral valve disease with AF. The primer to the INVICTUS study was a meta-analysis of four pivotal RCTs of DOAC in which patients with AF with valvular heart disease other than moderate to severe mitral stenosis were included [9]. The meta-analysis found DOAC to be an alternative to VKA with respect to efficacy and safety. Subsequently, the RIVER study found that rivaroxaban was not inferior to VKA for cardiovascular events, death, and bleeding in patients with mitral valve disease and AF undergoing bioprosthetic mitral valve implantation [10]. The study enrolled close to 1000 people across 50 sites in Brazil. Death from cardiovascular causes and thromboembolic events were lower with rivaroxaban. More supporting data came from a multicenter ENVISAGE AF TAVI trial, where >1400 patients undergoing transcatheter aortic valve replacement (TAVR) with AF were randomized to VKA and DOAC [11]. DOAC (edoxaban) was not inferior to VKA for the composite endpoints of death, myocardial infarction, stroke, valve thrombosis, systemic thromboembolism, and bleeding. The small RISE-MS study (*n* = 40) in patients with moderate-to-severe mitral stenosis with AF demonstrated that rivaroxaban was safe and efficacious compared to VKA over a 12-month follow-up [12]. Magnetic resonance imaging (MRI) detected cerebral infarcts were lower with rivaroxaban, while left atrial appendage thrombogenicity was similar in both arms. The DECISIVE study enrolled 120 valvular heart disease patients with AF and randomized them to dabigatran and warfarin [13]. The primary endpoint of stroke or silent brain ischemia (on MRI) was 13% lower with DOAC, but the results were not statistically significant. Building upon the success of these studies, INVICTUS was expected to deliver favorable outcomes for DOAC.

The INVICTUS trial results suggest that, compared with rivaroxaban, vitamin K antagonist therapy results in a lower rate of ischemic stroke among patients with rheumatic heart disease-associated atrial fibrillation and lower mortality due to vascular causes without significantly increasing the rate of major bleeding. Since a large number of patients were recruited from lower-income countries, including South Asia, the results are pertinent to our practice. The results of the INVICTUS trial support the current guidelines that recommend vitamin K antagonist therapy for the prevention of stroke in patients with rheumatic heart disease in whom atrial fibrillation develops [1,5].

Whether the INVICTUS trial was a failure of DOAC or a failure of the strategy needs to be analyzed further. Despite the young age of the population, the mortality rate was quite high (22%). The warfarin group had lower rates of sudden cardiac death and death due to pump failure (combined together—2/3rd of all deaths), which are unlikely to be related to anticoagulation in any manner. Hence, the lower mortality in the VKA group cannot be ascribed to the failure or inferiority of rivaroxaban. The positive efficacy results were largely driven by lower deaths with VKA rather than attenuated strokes, which is the primary presumed benefit and indication of oral anticoagulation. Hence, we cannot conclude with certainty that rivaroxaban did not perform as expected.

The significantly higher stroke rates with DOAC were associated with four times higher drug discontinuation rates. Of the patients who discontinued DOAC, many received VKA. This is contrary to what we expected in real-world scenarios. With single daily dosing, less food and drug interaction, and freedom from INR monitoring, rivaroxaban should have had better compliance than VKA. Moreover, the incidence of gastrointestinal side effects was higher with rivaroxaban than with apixaban or edoxaban. In the large ARISTOPHANES database, apixaban was clearly superior to rivaroxaban in terms of both efficacy and bleeding [14]. Even in the DOAC meta-analysis of valvular heart disease with AF excluding mitral stenosis, rivaroxaban had a higher incidence of major bleeding than other DOAC. Hence, whether the use of another DOAC with a more favorable safety profile could improve drug compliance and outcomes remains speculative.

**Figure 2:**
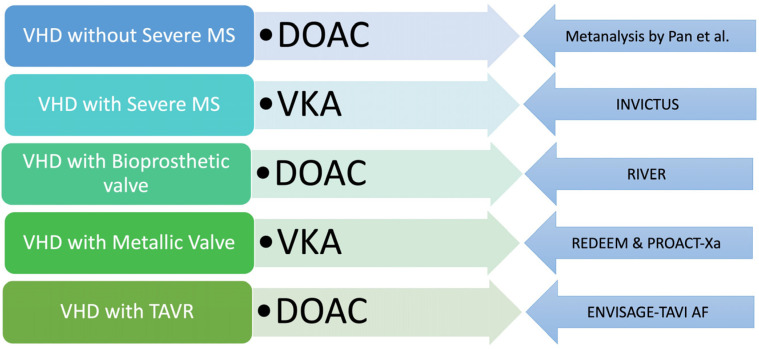
Current evidence-based approach to choice of oral anticoagulation in patients with AF with underlying VHD. The left panel depicts the subset of VHD, while the middle panel suggests the appropriate oral anticoagulant—DOAC/VKA. The right panel displays the evidence base for recommendation of oral anticoagulant in each scenario. [VHD, valvular heart disease; TAVR—transcatheter aortic valve replacement; MS—mitral stenosis; DOAC, direct oral anticoagulant; VKA—Vitamin K antagonist].

More negative data on DOAC in the setting of valvular heart disease have emerged from the PROACT-Xa trial, which was a study on apixaban in the setting of implantation of a metallic valve (the ON-X valve) but was stopped prematurely due to futility [15]. Previously, REDEEM failed to show the benefit of a DOAC (dabigatran) in the setting of a mechanical heart valve. However, since dabigatran is a direct thrombin inhibitor with 1:1 molar inhibition of thrombin, the use of upstream factor Xa inhibitors (rivaroxaban or apixaban) was proposed to be more beneficial in this setting, and the results of the PROACT-Xa study were eagerly awaited [16]. The failure of the INVICTUS trial along with the PROACT-Xa trial is a clear indication for VKA using oral anticoagulation in the setting of rheumatic mitral stenosis and metallic heart valves. With AF in the setting of valvular heart disease other than significant mitral stenosis and bioprosthetic valves (surgical or transcatheter), a DOAC can be used based on the patient/physician preference. Hence, appropriate clinical judgement based on contemporary evidence is needed to choose the right OAC for valvular heart disease with AF (Figure 2).

In this context, the results of the FRAIL-AF study are noteworthy [17]. The study aimed to answer an important question—whether elderly and frail patients with AF who are stable on VKA should be switched to DOAC given their potential for reduction in intracranial hemorrhage (ICH) and the high risk of ICH in this subset. Elderly and frail patients with AF on VKA were randomized to switch from VKA to DOAC or continue VKA therapy. Major bleeding or clinically relevant non-major bleeding at 12 months was hypothesized to be lower with the intervention (switch to DOAC). However, at the end of the study, bleeding was higher in the intervention arm, indicating that a routine switch from VKA to DOAC was not warranted. However, rivaroxaban was the DOAC used here again, as seen in the INVICTUS study. It remains to be seen whether this was a failure of the strategy or a failure of individual DOAC.

## 4. Future Directions

Few studies ongoing studies are comparing DOAC and VKA in valvular heart disease. The ERTEMIS (NCT05540587) study compared edoxaban 60 mg daily with warfarin for rheumatic mitral stenosis with AF [18]. The primary endpoint of the study was stroke/systemic embolism at 15 days follow up. Another ongoing trial (DAVID-MS; NCT04045093) is utilizing dabigatran in mitral stenosis with AF and comparing rates of stroke/systemic embolism at 1 year with warfarin [19]. The DIAMOND study (NCT05687448) enrolled patients with mechanical heart valves for oral anticoagulation with VKA or apixaban (5 mg BD) [20]. The NOTION 4 study (NCT06449469) evaluated the impact of DOAC on subclinical leaflet thickening on cardiac CT in patients undergoing TAVR [21]. DOACs are a heterogeneous group with respect to bleeding, as described above, but the evidence is indirect, as head-to-head studies of DOAC in AF are lacking. The COBRRA-AF study (NCT04642430) enrolled patients with AF and randomized them to receive rivaroxaban and apixaban in standard doses. The main endpoint was clinically relevant bleeding at 12 months [22].

Factor XI inhibitors represent a breakthrough in anticoagulation therapy, as they appear to dissociate thrombosis from bleeding [23,24,25]. Hence, these drugs offer the prospect of effective anticoagulation with minimal bleeding, which translates into practice from knowledge of hereditary factor XI deficiency leading to milder bleeding while offering protection from stroke/thromboembolism [24]. Multiple agents have successfully entered phase 3 studies in patients with AF and venous thromboembolism.

Early studies on novel anticoagulants targeting factor XI showed benefits in preventing venous thromboembolism in knee arthroplasty patients. Phase II studies are ongoing for cancer patients and hemodialysis patients with chronic kidney disease. The majority of clinical trials employing FXI inhibitors in humans are currently in phase 2, with each drug briefly outlined.

IONIS-FXI Rx/FXI-ASO is an antisense oligonucleotide (ASO) against Factor XI. The IONIS-FXI Rx/FXI-ASO molecule was the first molecule evaluated in phase II trials, showing superior efficacy in preventing venous thromboembolism in patients with total knee arthroplasty (FXI-ASO TKA) [26]. The 200 mg dose regimen was non-inferior, while the 300 mg dose was superior. The incidence of bleeding was comparable in both doses but lower than enoxaparin. Another phase II trial (NCT02553889) in ESRD patients showed a decline in FXI activity, with major bleeding events occurring in one patient [27]. A ligand-conjugated variant of IONIS-FXI Rx (Fesomersen) has greater efficacy and allows once-monthly treatment (RE-THINC ESRD) in ESRD patients undergoing hemodialysis [28].

Osocimab is a long-acting humanized monoclonal antibody against factor XIa. The FOXTROT trial compared the safety and efficacy of four different osocimab dosages to enoxaparin 40 mg once daily and apixaban 2.5 mg twice daily for preventing VTE in knee replacement patients [29]. Post-operative osocimab administration was non-inferior to enoxaparin, but 1.8 mg/kg dose was superior for preventing VTE at 10–13 days. The Osocimab arm had lower bleeding rates than the enoxaparin arm. The CONVERT trial enrolled patients with CKD and demonstrated that osocimab is generally well-tolerated in this population and corresponds to a reduced risk of bleeding [30].

Abelacimab is a humanized IgG1 monoclonal antibody that inhibits factor XI in its zymogen form and prevents factor XIIa and factor IIa from their activation. A trial (ANT-005 TKA) involving 412 patients undergoing total knee arthroplasty randomized them to either abelacimab (30 mg, 75 mg, 150 mg) or enoxaparin 40 mg subcutaneously once daily [31]. VTE rates were lower in the abelacimab group (13%, 5%, and 4%), while the 75 mg and 150 mg regimens were superior to enoxaparin. Non-major bleeding occurred in 2% of individuals receiving 30 mg or 75 mg abelacimab. The AZALEA-TIMI 71 trial evaluated the safety and efficacy of abelacimab in patients with atrial fibrillation (AF) at a moderate-to-high risk of stroke compared to rivaroxaban [32]. The study involved 1287 patients and found that both abelacimab dosages were superior to rivaroxaban 20 mg daily in decreasing bleeding episodes in individuals with AF and a high CHA2DS2-VASc score. Phase III trials are underway to evaluate the efficacy of abelacimab in preventing cancer-related thrombosis (NCT05171049; NCT05171) [33,34].

Xisomab is a recombinant antibody that selectively reduces factor XI activation through FXIIa feedback, with a differential increase in half-life with an increased dose. A randomized, placebo-controlled trial (NCT03612856) tested two doses of xisomab (0.25, 0.5 mg/kg) in 24 ESRD patients on hemodialysis [35]. The study found no significant bleeding, no significant time to hemostasis, and a single major bleeding event. However, there was a reduction in circuit occlusion rates requiring circuit exchange and decreased levels of thrombin-antithrombin complexes and C-reactive protein. A phase 2 prospective study (NCT04465760) evaluated the safety and efficacy of gruticibart, an anti-FXI monoclonal antibody, in 11 ambulatory cancer patients undergoing central line insertion [36]. The study found no significant catheter-associated thrombosis in the gruticibart group and no adverse events.

Asundexian is a direct inhibitor factor Xia. In the PACIFIC-AF study, Asundexian (20 mg or 50 mg) was compared with apixaban (5 mg) in non-valvular AF patients aged > 45 years [37]. The study enrolled 862 patients across 14 countries, and the primary end point was major or relevant non-major bleeding defined by ISTH criteria. There was a 67% reduction in bleeding with Asundexian (both doses combined) compared with apixaban at the end of the study, while side effects were similar to apixaban. The PACIFIC-AMI study compared three different dosages of Asundexian in patients with acute myocardial infarction [38]. The study involved 1600 patients randomized to three dosage groups (10 mg, 20 mg, and 50 mg), with Asundexian 50 mg showing over 90% inhibition of factor Xia levels. However, the efficacy outcome (composite of cardiovascular mortality, MI, stroke, or stent thrombosis) was identical in the maximum tested dose of Asundexian and placebo, suggesting that Asundexian 50 mg daily did not provide significant ischemic benefits compared to the placebo. The PACIFIC-STROKE study found that Asundexian, at varying doses, did not significantly reduce ischemic stroke and covert brain infarction compared to placebo, but it did reduce recurrent symptomatic strokes and TAIs in atherosclerosis patients [39]. The OCEANIC-AF trial tested asundexian against apixaban in atrial fibrillation patients at risk for stroke but was terminated early due to poor efficacy compared to the control arm [40].

Factor XI inhibitors outperformed enoxaparin in reducing VTE incidence and bleeding risk in phase II studies. However, higher doses did not reduce thrombotic events post MI, stroke, or TIA. Larger studies may establish their clinical importance in the future. Similarly, factor XII and XIII inhibitors are also in the pipeline. It will be interesting to examine the effects of these new-generation oral anticoagulants on rheumatic AF.

DOACs have emerged as a viable alternative to fill the unmet need with oral anticoagulation using VKA in AF/VTE patients. Although their ease of use and reduced bleeding events have made them popular, rheumatic mitral stenosis patients were majorly excluded from the seminal DOAC studies. The INVICTUS study was designed to evaluate the role of DOAC in rheumatic AF, but rivaroxaban failed to demonstrate superiority over VKA in this setting. Close to the heels of the INVICTUS trial, DOACs failed to prove superiority over VKA in AF, complicating the mechanical prosthetic valve in the PROACT-Xa study. Ongoing studies with contemporary DOACs and new generation OACs such as Factor XI inhibitors are expected to shed further light on the matter. The failure of DOACs in INVICTUS and PROACT-Xa studies has put an end to their unstoppable juggernaut. For patients of AF with metallic prosthetic valves, pregnancy, severe mitral stenosis, and antiphospholipid antibody syndrome, VKA’s are still the standard of care, and there is no need to retire them as of yet.

## 5. Conclusions

**Figure 3:**
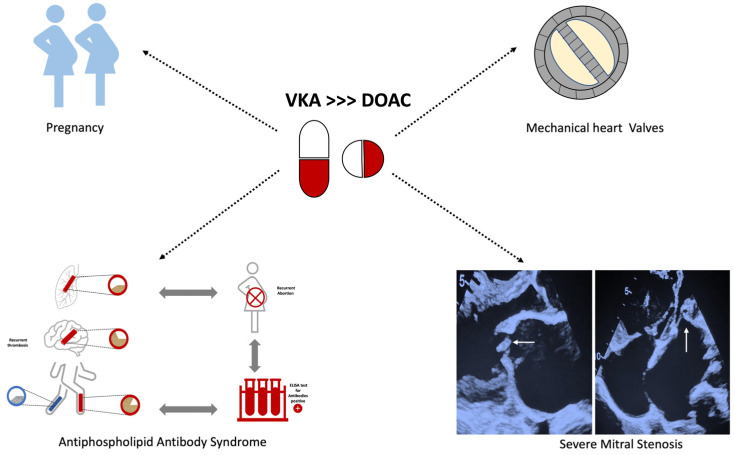
Current clinical scenario’s in AF patients where VKA’s would be still the first line agent for OAC. The upper right panel depicts a St. Jude’s bi-leaflet prosthetic valve. The lower right panel depicts the 2D transthoracic echocardiogram images in patients with severe rheumatic mitral stenosis. The lower left picture represents the triad of antiphospholipid antibody syndrome—recurrent abortions, arterial (red)/venous (blue) thrombosis, and a positive ELISA test for anti-cardiolipin/anti-B_2_-microglobulin antibody. [VKA—Vitamin K antagonist; DOAC—direct-acting anticoagulant; ELISA—enzyme-linked immunosorbent assay].

DOAC are preferred over VKA for stroke prevention in AF by major international guidelines, as they have been shown to reduce intracerebral hemorrhage in pivotal trials with equivalent efficacy. In addition, DOAC use has other practical advantages, such as amelioration of the need for INR monitoring, fewer drug interactions, and minimal food restrictions, resulting in wider acceptance across the globe. However, as patients with valvular AF were excluded from these studies, VKA are the oral anticoagulants of choice in this scenario of valvular AF, especially mitral stenosis, and the INVICTUS study failed to prove the efficacy of rivaroxaban in rheumatic mitral stenosis with AF. DOAC use was not associated with lower bleeding or stroke rates than VKA therapy. In contrast, death rates increased with the use of rivaroxaban. The higher drug discontinuation rate in the DOAC arm is perplexing, given the multiple advantages of DOAC. Whether substitution with alternate DOAC could have altered the results remains unanswered. On the other hand, the failure of apixaban in the PROACT-Xa study is another limitation to the use of DOAC in patients with mechanical heart valves. However, DOAC can still be utilized in patients with bioprosthetic valve implantation, transcatheter aortic valve replacement, and other native valve diseases with AF, except for moderate to severe mitral stenosis. Pending further studies, there is no current justification for DOAC prescription for AF with mitral stenosis and prosthetic valves (Figure 3). Factor XI inhibitors are currently on the anvil and hold great promise for the future. Ongoing studies of DOAC in the valvular heart are expected to shed further light on their role.

## Figures and Tables

**Table 1:** The “Pros” and “Cons” of DOAC use for oral anticoagulation for AF. [DOAC, direct oral anticoagulant; VKA—vitamin K antagonist; ICH, intracerebral hemorrhage].

Advantage	Disadvantage
Predictable pharmacodynamics and pharmacokinetics.	Standardized tests for monitoring of DOAC activity are not easily available, when it is necessary in liver and kidney injury.
Low drug-drug and food interactions.	Currently lack a universal antidote
No dietary restriction.	High cost (in some countries)
Rapid onset and offset, short half life.	Experience limited to a decade
No need of regular laboratory monitoring unlike VKA	Needed dose modification in renal dysfunction
Wide therapeutic window.	Superiority against VKA for efficacy is yet to be achieved
DOACs can be started without LMWH due to their rapid onset.	Not indicated in pregnancy
Low rates of ICH (50% reduction)	

## References

[1] Van Gelder I.C., Rienstra M., Bunting K.V., Casado-Arroyo R., Caso V., Crijns H.J.G.M., De Potter T.J.R., Dwight J., Guasti L., Hanke T. (2024). 2024 ESC Guidelines for the management of atrial fibrillation developed in collaboration with the European Association for Cardio-Thoracic Surgery (EACTS). Eur. Heart J..

[2] Ruff C.T., Giugliano R.P., Braunwald E., Hoffman E.B., Deenadayalu N., Ezekowitz M.D., Camm A.J., Weitz J.I., Lewis B.S., Parkhomenko A. (2014). Comparison of the efficacy and safety of new oral anticoagulants with warfarin in patients with atrial fibrillation: A meta-analysis of randomized trials. Lancet.

[3] Connolly S.J., Karthikeyan G., Ntsekhe M., Haileamlak A., El Sayed A., El Ghamrawy A., Damasceno A., Avezum A., Dans A.M.L., Gitura B. (2022). Rivaroxaban for rheumatic heart disease-associated atrial fibrillation. N. Engl. J. Med..

[4] Zimetbaum P. (2017). Atrial fibrillation. Ann. Intern. Med..

[5] Joglar J.A., Chung M.K., Armbruster A.L., Benjamin E.J., Chyou J.Y., Cronin E.M., Deswal A., Eckhardt L.L., Goldberger Z.D., Gopinathannair R. (2024). 2023 ACC/AHA/ACCP/HRS guidelines for the diagnosis and management of atrial fibrillation: A report of the American College of Cardiology/American Heart Association Joint Committee on Clinical Practice Guidelines. Circulation.

[6] Mekaj Y.H., Mekaj A.Y., Duci S.B., Miftari E.I. (2015). New oral anticoagulants: Their advantages and disadvantages compared with vitamin K antagonists in the prevention and treatment of patients with thromboembolic events. Ther. Clin. Risk Manag..

[7] Negi P.C., Sondhi S., Asotra S., Mahajan K., Mehta A. (2019). Current status of rheumatic heart disease in India. Indian. Heart J..

[8] Karthikeyan G., Connolly S.J., Yusuf S. (2020). Overestimation of Stroke Risk in Rheumatic Mitral Stenosis and the Implications for Oral Anticoagulation. Circulation.

[9] Pan K.L., Singer D.E., Ovbiagele B., Wu Y.L., Ahmed M.A., Lee M. (2017). Effects of Non-Vitamin K Antagonist Oral Anticoagulants Versus Warfarin in Patients with Atrial Fibrillation and Valvular Heart Disease: A Systematic Review and Meta-Analysis. J. Am. Heart Assoc..

[10] Guimarães H.P., Lopes R.D., de Barros ESilva P.G.M., Liporace I.L., Sampaio R.O., Tarasoutchi F., Hoffmann-Filho C.R., de Lemos Soares Patriota R., Leiria T.L.L., Lamprea D. (2020). Rivaroxaban in Patients with Atrial Fibrillation and a Bioprosthetic Mitral Valve. N. Engl. J. Med..

[11] Van Mieghem N.M., Unverdorben M., Hengstenberg C., Möllmann H., Mehran R., López-Otero D., Nombela-Franco L., Moreno R., Nordbeck P., Thiele H. (2021). Edoxaban versus vitamin K antagonists for atrial fibrillation after TAVR. N. Engl. J. Med..

[12] Sadeghipour P., Pouraliakbar H., Parsaee M., Shojaeifard M., Farrashi M., Jamal Khani S., Tashakori Beheshti A., Rostambeigi S., Ebrahimi Meimand S., Firouzi A. (2022). RIvaroxaban in mitral stenosis (RISE MS): A pilot randomized clinical trial. Int. J. Cardiol..

[13] Cho M.S., Kim M., Lee S.A., Lee S., Kim D.H., Kim J., Song J.M., Nam G.B., Kim S.J., Kang D.H. (2022). Comparison of Dabigatran Versus Warfarin Treatment for Prevention of New Cerebral Lesions in Valvular Atrial Fibrillation. Am. J. Cardiol..

[14] Lip G.Y.H., Keshishian A., Li X., Hamilton M., Masseria C., Gupta K., Luo X., Mardekian J., Friend K., Nadkarni A. (2018). Effectiveness and Safety of Oral Anticoagulants Among Nonvalvular Atrial Fibrillation Patients. Stroke.

[15] Wang T.Y., Svensson L.G., Wen J., Vekstein A., Gerdisch M., Rao V.U., Moront M., Johnston D., Lopes R.D., Chavez A. (2023). Apixaban or Warfarin in Patients with an On-X Mechanical Aortic Valve. NEJM Evid..

[16] Narain V.S., Pradhan A., Bhandari M., Banerjee P.S., Das M.K., Roy D. (2021). Mechanical Prosthetic valve on Oral Anticoagulants—How to improve safety. Cardiology Update Book 2021—Cardiological Society of India.

[17] Joosten L.P., van Doorn S., van de Ven P.M., Köhlen B.T., Nierman M.C., Koek H.L., Hemels M.E., Huisman M.V., Kruip M., Faber L.M. (2023). Safety of Switching from Vitamin K Antagonist to Non-Vitamin K Antagonist Oral Anticoagulant in Frail Older Patients with Atrial Fibrillation: Results of the Frail-AF Randomized Control Trial. Circulation.

[18] Efficacy and Safety of Edoxaban in Patients with Atrial Fibrillation and Mitral Stenosis (ERTEMIS). https://clinicaltrials.gov/study/NCT05540587.

[19] Zhou M., Chan E.W., Hai J.J., Wong C.K., Lau Y.M., Huang D., Lam C.C., Tam C.C.F., Wong Y.T.A., Yung S.Y.A. (2020). Protocol, rationale and design of DAbigatran for Stroke PreVention in Atrial Fibrillation in MoDerate or Severe Mitral Stenosis (DAVID-MS): A randomised, open-label study. BMJ Open.

[20] DIrect Oral Anticoagulation and mechaNical Aortic Valve (DIAMOND). https://clinicaltrials.gov/study/NCT05687448.

[21] The Nordic Aortic Valve Intervention Trial 4 (NOTION-4). https://clinicaltrials.gov/study/NCT06449469.

[22] COmparison of Bleeding Risk Between Rivaroxaban and Apixaban in Patients with Atrial Fibrillation (COBRRA-AF). https://clinicaltrials.gov/study/NCT04642430.

[23] Harrington J., Piccini J.P., Alexander J.H., Granger C.B., Patel M.R. (2023). Clinical Evaluation of Factor XIa Inhibitor Drugs: JACC Review Topic of the Week. J. Am. Coll. Cardiol..

[24] Preis M., Hirsch J., Kotler A., Zoabi A., Stein N., Rennert G., Saliba W. (2017). Factor XI deficiency is associated with lower risk for cardiovascular and venous thromboembolism events. Blood.

[25] De Caterina R., Prisco D., Eikelboom J.W. (2023). Factor XI inhibitors: Cardiovascular perspectives. Eur. Heart J..

[26] Büller H.R., Bethune C., Bhanot S., Gailani D., Monia B.P., Raskob G.E., Segers A., Verhamme P., Weitz J.I. (2015). Factor XI Antisense Oligonucleotide for Prevention of Venous Thrombosis. N. Engl. J. Med..

[27] Walsh M., Bethune C., Smyth A., Tyrwhitt J., Jung S.W., Yu R.Z., Wang Y., Geary R.S., Weitz J., Bhanot S. (2021). Phase 2 Study of the Factor XI Antisense Inhibitor IONIS-FXIRx in Patients with ESRD. Kidney Int. Rep..

[28] Winkelmayer W.C., Lensing A.W.A., Thadhani R.I., Mahaffey K.W., Walsh M., Pap Á.F., Willmann S., Thelen K., Hodge S., Solms A. (2024). A Phase II randomized controlled trial evaluated antithrombotic treatment with fesomersen in patients with kidney failure on hemodialysis. Kidney Int..

[29] Weitz J.I., Bauersachs R., Becker B., Berkowitz S.D., Freitas M.C., Lassen M.R., Metzig C., Raskob G.E. (2020). Effect of Osocimab in Preventing Venous Thromboembolism Among Patients Undergoing Knee Arthroplasty: The FOXTROT Randomized Clinical Trial. JAMA.

[30] Weitz J.I., Tankó L.B., Floege J., Fox K.A.A., Bhatt D.L., Thadhani R., Hung J., Pap Á.F., Kubitza D., Winkelmayer W.C. (2024). Anticoagulation with osocimab in patients with kidney failure undergoing hemodialysis: A randomized phase 2 trial. Nat. Med..

[31] Verhamme P., Yi B.A., Segers A., Salter J., Bloomfield D., Büller H.R., Raskob G.E., Weitz J.I., ANT-005 TKA Investigators (2020). Abelacimab for Prevention of Venous Thromboembolism. N. Engl. J. Med..

[32] Kumbhani D.J. A Multicenter, RandomiZed, Active-ControLled Study to Evaluate the Safety and Tolerability of Two Blinded Doses of Abelacimab Compared with Open-Label Rivaroxaban in Patients with Atrial Fibrillation—AZALEA-TIMI 78. In: American College of Cardiology. [Internet]. https://www.acc.org/Latest-in-Cardiology/Clinical-Trials/2023/11/10/22/46/azalea-timi-71.

[33] A Study Comparing Abelacimab to Apixaban in the Treatment of Cancer-Associated VTE (ASTER). https://clinicaltrials.gov/study/NCT05171049.

[34] A Study Comparing Abelacimab to Dalteparin in the Treatment of Gastrointestinal/Genitourinary Cancer and Associated VTE (MAGNOLIA). https://clinicaltrials.gov/study/NCT05171075.

[35] Lorentz C.U., Tucker E.I., Verbout N.G., Shatzel J.J., Olson S.R., Markway B.D., Wallisch M., Ralle M., Hinds M.T., McCarty O.J.T. (2021). The contact activation inhibitor AB023 in heparin-free hemodialysis: Results of a randomized phase 2 clinical trial. Blood.

[36] Pfeffer M.A., Kohs T.C.L., Vu H.H.L., Jordan K.R., Wang J.S.H., Lorentz C.U., Tucker E.I., Puy C., Olson S.R., DeLoughery T.G. (2024). Factor XI Inhibition for the Prevention of Catheter-Associated Thrombosis in Patients with Cancer Undergoing Central Line Placement: A Phase 2 Clinical Trial. Arterioscler. Thromb. Vasc. Biol..

[37] Piccini J.P., Caso V., Connolly S.J., Fox K.A.A., Oldgren J., Jones W.S., A Gorog D., Viethen T., Neumann C., Mundl H. (2022). Safety of the oral factor XIa inhibitor asundexian compared with apixaban in patients with atrial fibrillation (PACIFIC-AF): A multicentre, randomised, double-blind, double-dummy, dosefinding phase 2 study. Lancet.

[38] Rao S.V., Kirsch B., Bhatt D.L., Budaj A., Coppolecchia R., Eikelboom J., James S.K., Jones W.S., Merkely B., Keller L. (2022). A Multicenter, Phase 2, Randomized, Placebo-Controlled, Double-Blind, Parallel-Group, Dose-Finding Trial of the Oral Factor XIa Inhibitor Asundexian to Prevent Adverse Cardiovascular Outcomes After Acute Myocardial Infarction. Circulation.

[39] Shoamanesh A., Mundl H., Smith E.E., Masjuan J., Milanov I., Hirano T., Agafina A., Campbell B., Caso V., Mas J.-L. (2022). Factor XIa inhibition with asundexian after acute non-cardioembolic ischaemic stroke (PACIFIC-Stroke): An international, randomised, double-blind, placebo-controlled, phase 2b trial. Lancet.

[40] Piccini J.P., Patel M.R., Steffel J., Ferdinand K., Van Gelder I.C., Russo A.M., Ma C.-S., Goodman S.G., Oldgren J., Hammett C. (2024). Asundexian Versus Apixaban in Patients with Atrial Fibrillation. N. Engl. J. Med..

